# Double Chambered Left Ventricle in a 56-YOF scheduled for transcatheter aortic valve implantation (TAVI) planning incidentally detected by CTA

**DOI:** 10.1007/s10554-024-03181-0

**Published:** 2024-07-05

**Authors:** Pietro G. Lacaita, Fabian Barbieri, Gerlig Widmann, Gudrun M. Feuchtner

**Affiliations:** 1 Department of Radiology, Innsbruck Medical University, Anichstrasse 35, Innsbruck, 6020 Austria; 2 Department of Cardiology, Angiology and Intensive Care Medicine, Deutsches Herzzentrum der Charité, Hindenburgdamm 30, 12203 Berlin, Germany

**Keywords:** Computed tomography, Double-chambered left ventricle, Congenital heart diease, Cardiac

A 56-years-old female with severe aortic stenosis was referred to cardiac computed tomography angiography (CTA) for planning of transcatheter aortic valve implantation (TAVI). She had undergone surgical closure of a membranous ventricular septal defect (VSD), and pulmonary banding with debanding 8 years later. Echocardiography reported muscular VSDs with hypertrabeculations (“swiss-cheese”-pattern) and almost-total closure with possible minimal residual shunt.

Cardiac CTA showed an unusual left ventricle (LV) consisting of two chambers with apexes oriented parallel anteriorly oblique (Fig. 1), and excessive LV-hypertrabeculation apical and midventricular septal (*mid panel*) mimicking a muscular VSD midseptal. TAVI was deferred to avoid the risk of wire trapping and LV-perforation.

**Fig. 1:**
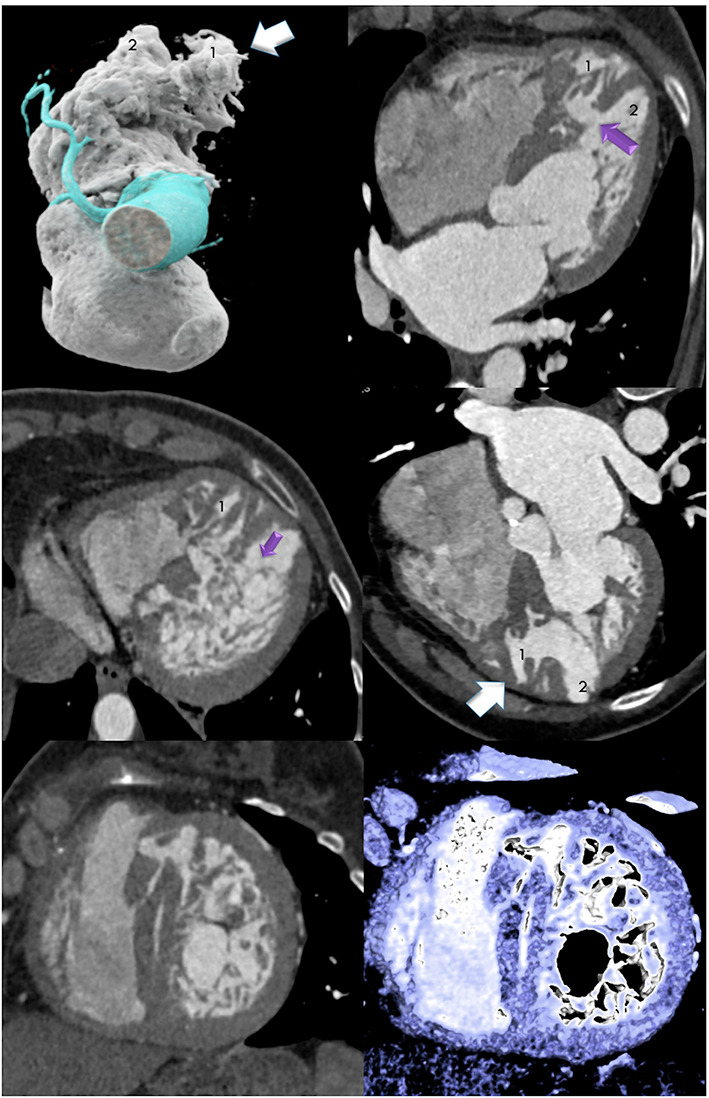
Double Chambered Left Ventricle (DCLV) (upper panel) with an accessory left ventricular (LV) chamber (1) adjacent to the normal LV apex (2). Cinematric 3D- Volume rendering technique (VRT) (left) and multiplanar reformation (MPR) axial image (upper panel, left) with excessive LV- Hypertrabeculation mimicking muscular ventricular septal defect (VSD) (purple arrow, right upper panel) Hypertrabeculation basal (purple arrow, mid panel, left), 3-Chamber view (mid-panel, right) and short axis view midseptal (lower panel)

She underwent mechanical aortic valve replacement (21 mm On-X™ prosthesis) and patch repair of the residual pulmonary stenosis. The small residual VSD reported by echocardiography could not be located intraprocedural. 1 year follow-up was uneventful.

Double-chambered left ventricle (DCLV) is extremely rare (estimated prevalence 0.04–0.42%)– although the exact prevalence is unknown and most commonly involving the right ventricle, while the incidence left is even scarcer with only few cases reported worldwide.

DCLV is characterized by the presence of two LV-chambers, oriented in parallel and must be distinguished from a LV-diverticulum by the presence of an additional contractile septum.

## Electronic supplementary material

Below is the link to the electronic supplementary material.

Supplementary Material 1

## Data Availability

No datasets were generated or analysed during the current study.

